# Comparative study of hyperpolarization-activated currents in pulmonary vein cardiomyocytes isolated from rat, guinea pig, and rabbit

**DOI:** 10.1186/s12576-020-00736-3

**Published:** 2020-02-11

**Authors:** Daichi Takagi, Yosuke Okamoto, Takayoshi Ohba, Hiroshi Yamamoto, Kyoichi Ono

**Affiliations:** 1 Department of Cardiovascular Surgery, Akita University Graduate School of Medicine, Hondo 1-1-1, Akita, 010-8543 Japan; 2 Department of Cell Physiology, Akita University Graduate School of Medicine, Hondo 1-1-1, Akita, 010-8543 Japan

**Keywords:** Hyperpolarization-activated cation current, Hyperpolarization-activated Cl^−^ current, Pulmonary vein, Automaticity, Atrial fibrillation

## Abstract

Pulmonary vein (PV) cardiomyocytes have the potential to generate spontaneous activity, in contrast to working myocytes of atria. Different electrophysiological properties underlie the potential automaticity of PV cardiomyocytes, one being the hyperpolarization-activated inward current (*I*_h_), which facilitates the slow diastolic depolarization. In the present study, we examined pharmacological characteristics of the *I*_h_ of PV cardiomyocytes in rat, guinea pig and rabbit. The results showed that guinea pig and rat PV cardiomyocytes possessed sizeable amplitudes of the *I*_h_, and the *I*_h_ of guinea pig was suppressed by Cs^+^, a blocker of the hyperpolarization-activated cation current. However, the *I*_h_ of rat was not suppressed by Cs^+^, but by Cd^2+^, a blocker of the Cl^−^ current. The current density of the *I*_h_ of rabbit PV cardiomyocytes was significantly smaller than those of other species. This suggests that the ion channels that carry the *I*_h_ of PV cardiomyocytes differ among the animal species.

## Introduction

The striated myocardium extends from the left atrium (LA) into the ostia of the pulmonary veins (PV), and ectopic activity in the PV myocardium often accounts for initiation and maintenance of atrial fibrillation (AF), the most frequent sustained arrhythmia encountered in clinical practice [1]. During development, PV cardiomyocytes are differentiated from mesenchymal cells surrounding the developing venous pole, and the ectopic pacemaker fate is promoted under the influence of the antagonistic action of Shox2, a member of the homeobox family of genes, on Nkx2-5 [2, 3]. Electrophysiological experiments demonstrated various types of spontaneous activity in PV cardiomyocytes in animal studies [4]. These include sinoatrial node-like spontaneous action potentials [5, 6], digitalis-induced arrhythmia [7, 8], noradrenaline-induced automaticity [9–11], stretch-induced automaticity [12] and pacing-induced spontaneous activity [6, 13, 14]. Such arrhythmogenic activity depends, in part, upon their distinct electrophysiological characteristics, i.e., the PV myocardium in general has a less negative resting membrane potential when compared with the atrial myocardium. In addition, it has been reported that the hyperpolarization-activated inward current (*I*_h_), which is activated during diastole, facilitates the automaticity of the PV myocardium. One of *I*_h_ is the hyperpolarization-activated cation current (*I*_f_), which acts as a pacemaker current in normal pacemaker cells of the sinoatrial node [15] and exists in PV cardiomyocytes of the dog and rabbit [5, 16, 17]. The *I*_f_ is a mixed current of Na^+^ and K^+^, and is characterized by its relatively slow time course of activation on hyperpolarization [15]. However, we previously found another *I*_h_ which is carried by Cl^−^ in rat PV cardiomyocytes [11]. We designated this current as the hyperpolarization-activated Cl^−^ current (*I*_Cl,h_). The *I*_Cl,h_ showed a similar slow time course of activation with *I*_f_, but was insensitive to 5 mM Cs^+^, a blocker for *I*_f_, and the removal of external K^+^ or Na^+^ had no effect on the current. The reversal potential was near − 20 mV at the 40-mM [Cl^−^]_i_ and 148.9-mM [Cl^−^]_o_ conditions, and was shifted to depolarized potentials by increasing [Cl^−^]_i_ or by decreasing [Cl^−^]_o_. These characteristics are totally different from those of an *I*_f_ or a K^+^ current (*I*_KH_) [18], which was attributed to the *I*_h_ in dog PV cardiomyocytes. Furthermore, reagents that attenuate the Cl^−^ current suppressed the norepinephrine-induced automaticity of rat PV cardiomyocytes, indicating a functional role of the Cl^−^ current in the automaticity of the PV myocardium. However, little is known whether the *I*_Cl,h_ exists in other experimental animal species. In the present study, therefore, we examined the species differences of the *I*_h_ of PV cardiomyocytes isolated from rat, guinea pig and rabbit under identical experimental conditions.

## Materials and methods

### Cell isolation

The protocols used in this study were approved by the Animal Ethics Committee of the Akita University School of Medicine, Japan. Cell isolation procedures were essentially similar to those reported previously [10, 11]. Male Wister rats (8–12 weeks old, 300–400 g) and male guinea pigs (5–10 weeks old, 400–700 g) were anesthetized by intraperitoneal injection of pentobarbital sodium (100 and 120 mg/kg for rats and guinea pigs, respectively). Male rabbits (10–16 weeks old, 2.0–3.0 kg) were anesthetized by intravenous injection of xylazine (5 mg/kg) and ketamine (35 mg/kg). After checking suppression of the nociceptive reflex, the chest of each animal was opened under artificial respiration and the aorta was cannulated in situ to perfuse the coronary arteries. The heart and lung were excised en bloc, mounted on a Langendorff apparatus, then perfused sequentially with the following buffers: (1) normal Tyrode’s solution for a few minutes; (2) nominally Ca^2+^-free Tyrode’s solution for 5 min; and (3) Ca^2+^-free Tyrode’s solution containing 0.05% collagenase (Wako Pure Chemical, Osaka, Japan) for 30 min in rats and guinea pigs. As for rabbits, collagenase alone was not effective to digest the tissue and obtain isolated cardiomyocytes, and therefore 0.07% collagenase and 0.005% elastase (Wako Pure Chemical, Osaka, Japan) were used for the enzyme treatment. We then trimmed off the soft tissue containing the vagal nerve, adipose tissue and the pulmonary artery. The LA and PV were then excised from the digested block, minced in high K^+^, low Cl^−^ solution and agitated to dissociate the cells. The cell suspension was stored at 4 °C for later use.

### Solutions

The composition of the normal Tyrode’s solution (mM) was: NaCl 136.9, KCl 5.4, CaCl_2_ 1.8, MgCl_2_ 0.5, NaH_2_PO_4_ 0.33, HEPES 5.0, and glucose 5.5 (pH 7.4 with NaOH). The high K^+^, low Cl^−^ solution for cell storage contained (mM): l-glutamic acid 70, KOH 70, KCl 30, KH_2_PO_4_, MgCl_2_ 1, taurine 20, glucose 10, EGTA 0.3, and HEPES 10 (pH 7.4 with KOH). The internal solution for the conventional whole-cell clamp experiments contained (mM): KOH 120, aspartic acid 80, Mg-ATP 5, KCl 20, HEPES 5, EGTA 5, and GTP-Na_2_ 0.1 (pH 7.2 with aspartic acid). For the perforation patch-clamp recording, the pipette solution was composed of (mM) KOH 110, aspartic acid 110, KCl 30, NaCl 10, HEPES 5, and EGTA 10 (pH 7.2 with KOH)﻿, and amphotericin B 0.2 mg/mL was added.

To block the l-type Ca^2+^ current (*I*_CaL_), 0.3 μM nisoldipine was added to the normal Tyrode’s solution. BaCl_2_, CsCl and CdCl_2_ were used to block the inward rectifier K^+^ current (*I*_K1_), *I*_f_ and *I*_Cl,h_, respectively, in the present study. They were dissolved in distilled water as 1 M stock solution and added to the normal Tyrode’s solution to obtain the final concentrations described in the text.

### Electrophysiological analysis

The whole-cell patch-clamp method was used for recording membrane potentials and currents (patch-clamp amplifier Axopatch 1D or Axopatch 200B, Molecular Devices, Chicago, IL, USA). Borosilicate glass electrodes had tip resistances between 2.0 and 5.0 MΩ when filled with internal solution. Action potentials were recorded using the perforated patch-clamp technique at 35 ± 0.5 °C. Membrane currents were recorded under voltage-clamp conditions at 35–36 °C. Pulse protocols and data acquisition and storage were accomplished with CLAMPEX (Molecular Devices, Chicago, IL, USA). The cell membrane capacitance (C_m_) was determined by applying a 30-ms hyperpolarizing voltage-clamp step from a holding potential of − 40 mV to − 50 mV, then dividing the time-integral of the capacitive current by the voltage step. All patch-clamp data were analyzed using IGOR software (version 7.0, Wavemetrics, Portland, OR, USA).

### Statistical analysis

Data are expressed as mean ± standard error. Statistical significance was evaluated using Student’s *t* test or one-way ANOVA followed by a post hoc test with Bonferroni correction. A *p* value less than 0.05 was considered statistically significant. The number of cells (*n*) used in each experiment is indicated in the figures or text.

## Results

### Action potential and whole-cell currents

**Fig. 1:**
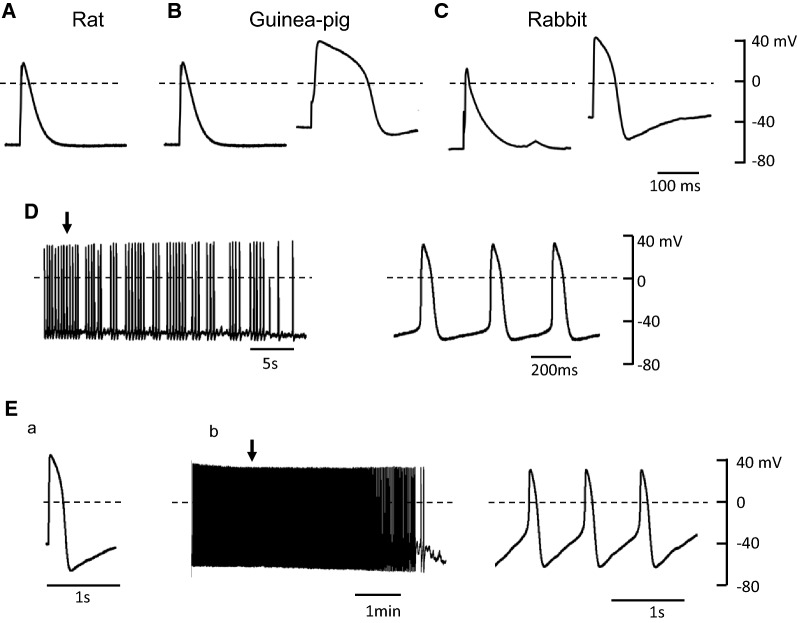
Representative action potentials recorded in PV cardiomyocytes of rat (**A**), guinea pig (**B**), and rabbit (**C**). Spontaneous action potentials recorded in PV cardiomyocytes of guinea pig (**D**). The action potential indicated by the arrow is shown in an expanded time scale in the right panel. Typical tracings recorded from the PV cardiomyocytes of rabbit are an elicited action potential (Ea) and subsequent spontaneous electrical activity (Eb) after the train stimulation at a pacing cycle length of 2 s. The action potential indicated by the arrow is shown in an expanded time scale at the right panel. Dashed lines indicate 0 mV

Figure  1A shows representative traces of evoked action potential recorded in PV cardiomyocytes of rat, guinea pig, and rabbit. In PV cardiomyocytes of rat, the resting potential was − 71 ± 2 mV (*n* = 9) and spontaneous activity was not observed. However, the spontaneous action potentials were recorded in 1 out of 10 and in 5 out of 9 PV cardiomyocytes of guinea pig and rabbit, respectively. The quiescent PV cardiomyocyte in guinea pig and rabbit PV showed a resting potential of − 58.4 ± 4.8 mV (*n* = 10) and − 40.1 ± 4.2 mV (*n* = 9), respectively, with variable AP durations as shown in Fig. 1B, C. The spontaneous action potential observed in guinea pig PV cardiomyocytes is shown in Fig. 1D, where slow depolarization is seen during diastole. For rabbit, spontaneous activity was observed in 4 of 9 cells and, even in quiescent cells, a train stimulation at a pacing cycle length of 2 s successfully triggered a spontaneous activity (Fig. 1E). All these findings are consistent with previous findings that PV cardiomyocytes have the potential to generate spontaneous activity in various mammalian species

**Fig. 2:**
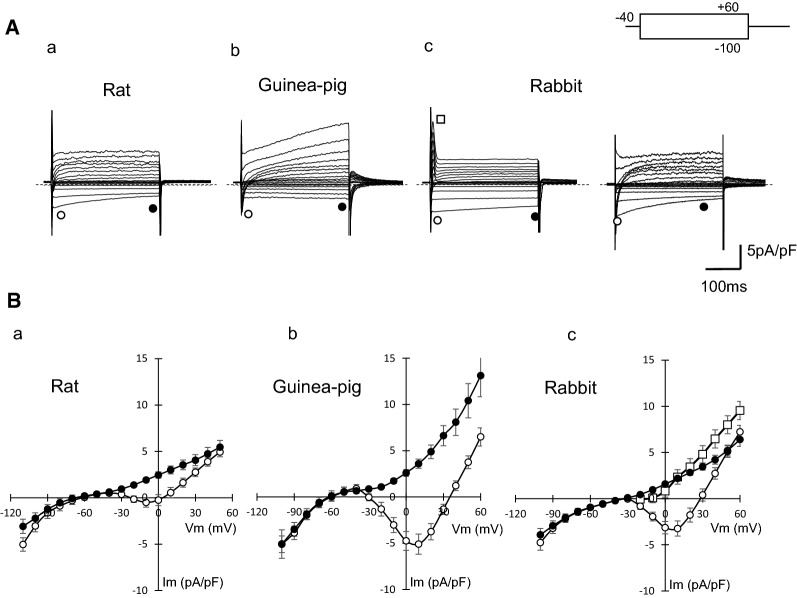
Comparison of membrane currents and *I–V* relationships of isolated PV cardiomyocytes from rat, guinea pig, and rabbit. **A** Current traces were obtained from PV cardiomyocytes of rat (**a**), guinea pig (**b**), and rabbit (**c**) in a normal Tyrode’s solution. Traces shown were obtained by applying 500-ms depolarizing or hyperpolarizing pulses from a holding potential of − 40 mV to a test potential from − 100 mV to + 60 mV. In PV cardiomyocytes of rabbit, two obviously different families of currents were detected, where some cells possessed transient outward current (left) and others did not (right). Dashed lines indicate the zero current level. **B***I–V* relationships for the initial current (open circles) and the current near the end of the pulses (filled circles) in rat (**a**), guinea pig (**b**), and rabbit (**c**). A transient outward current was shown only in rabbit (open squares)

Figure 2 shows whole-cell currents of PV cardiomyocytes of rat, guinea pig, and rabbit recorded in a normal Tyrode’s solution. Square pulses of 300 ms were applied from − 40 mV to various potentials ranging between − 100 mV and + 60 mV. In all species, activation of the *I*_CaL_ was followed by a delayed rectifier K^+^ current in response to depolarization, and the *I*_K1_ was predominant on hyperpolarization. In rabbit preparations, some cells showed a significant amplitude of transient outward currents (*I*_to_) upon depolarization (18 of 21 cells) (Fig. 2Ac, left panel), and others did not (Fig. 2Ac, right panel). The action potential of rabbit PV cardiomyocytes, which had no *I*_to_, showed less negative resting membrane and spontaneous electrical activity was recorded after train stimulation at a pacing cycle length of 2 s. The *C*_m_ of rat PV cardiomyocytes was 191.3 ± 23.0 (*n* = 20), which was significantly larger than those of guinea pig (63.7 ± 4.7 pF, *n* = 23) and rabbit (71.6 ± 7.4 pF, *n* = 30). The variable cell size and relatively larger *C*_m_ value of rat PV cardiomyocytes were consistent with our previous study [10].

### Time-dependent *I*_h_ in rat and guinea pig PV

**Fig. 3:**
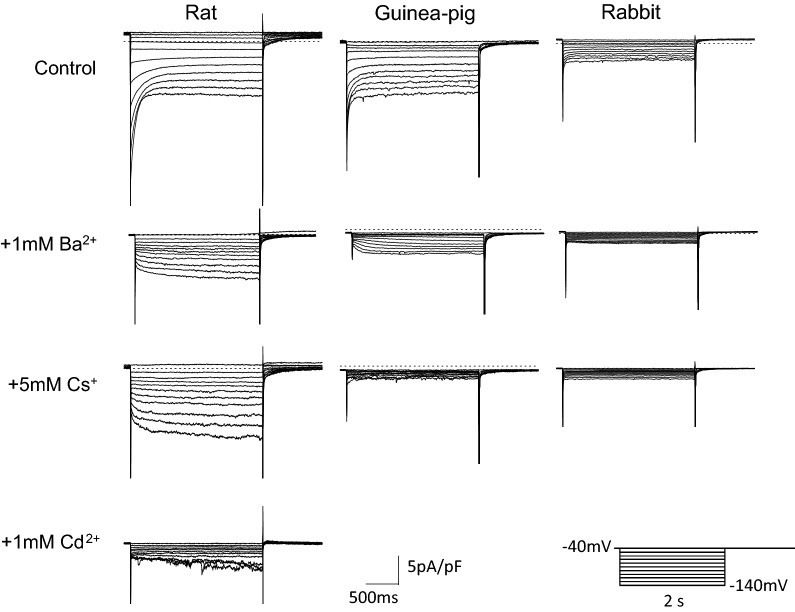
*I*_h_ in rat, guinea pig, and rabbit PV cardiomyocytes. Recordings from PV cardiomyocytes of rat (left), guinea pig (middle), and rabbit (right). Recordings in each animal were obtained in the same cell with 2-s hyperpolarizing pulses from − 40 mV to − 140 mV in 10 mV steps. Representative current traces were obtained in Tyrode solution containing 0.3 μM nisoldipine (top row). Then, the same pulse protocol was applied after addition of 1 mM Ba^2+^ (2nd row), 5 mM Cs^+^ (3rd row) and 1 mM Cd^2+^ (bottom row). The dashed line indicates 0 current level

**Fig. 4:**
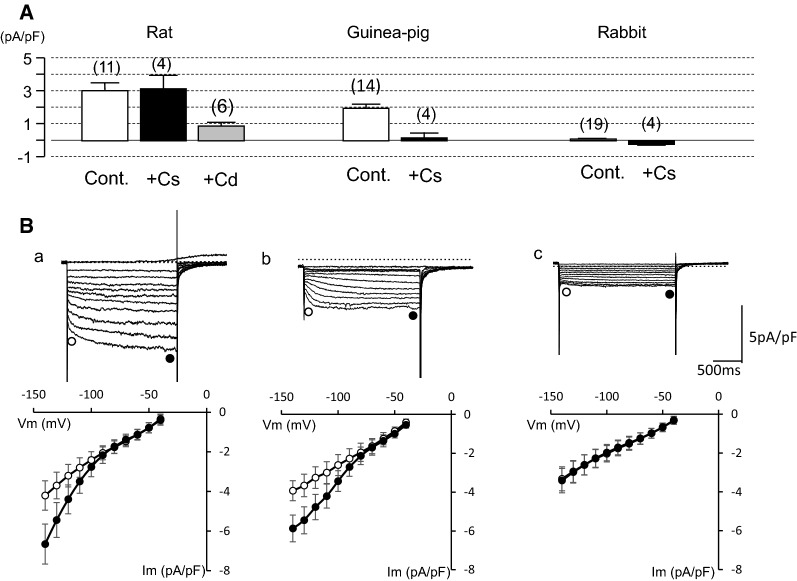
Comparison of the *I*_h_ in PV cardiomyocytes from rat, guinea pig, and rabbit. **A** The amplitude of the I_h_ in rat (left), guinea pig (middle), and rabbit (right) PV cardiomyocytes was measured at − 140 mV in the control and in the presence of either 5 mM Cs^+^ or 1 mM Cd^2+^. **B** The current amplitudes were measured at the beginning and near the end of the test pulse of PV cardiomyocytes in rat (left), guinea pig (middle), and rabbit (right)

In the experiment shown in Fig. 3, we attempted to record the *I*_h_. Upon hyperpolarizing voltage steps of 2 s each from − 40 mV to various potentials, PV cardiomyocytes showed instantaneous current jumps followed by a rapid decay (Fig. 3, upper panel). This current was inhibited by 1 mM Ba^2+^, indicating the inwardly rectifying K^+^ current (*I*_K1_) and the acetylcholine-activated K^+^ current. After inhibition of these K^+^ currents, a time-dependent *I*_h_ was observed in 55% (12 out of 22 cells) of rat PV cardiomyocytes and in 88% (14 out of 16 cells) of guinea pig ones. In rabbit PV cells, we failed to detect time-dependent currents (19 cells, Fig. 3, middle upper panel). The *I*_h_ of rat PV was not suppressed by 5 mM Cs^+^ and was suppressed by 1 mM Cd^2+^, while it was suppressed by 5 mM Cs^+^ but not by 1 mM Cd^2+^ in guinea pig PV. This finding was confirmed in six other cells in rat and in four other cells in guinea pig (Fig. 4A). The *I*_h_ amplitude at − 140 mV in Tyrode’s solution containing nisoldipine and 1 mM Ba^2+^ was − 3.1 ± 0.4 pA/pF in rat, − 1.9 ± 0.2 pA/pF in guinea pig, and − 0.09 ± 0.04 pA/pF in rabbit (Fig. 4A). These findings indicate that the *I*_h_ of rat PV is chiefly derived from *I*_Cl,h_, and the current of guinea pig consists of *I*_f_.

**Fig. 5:**
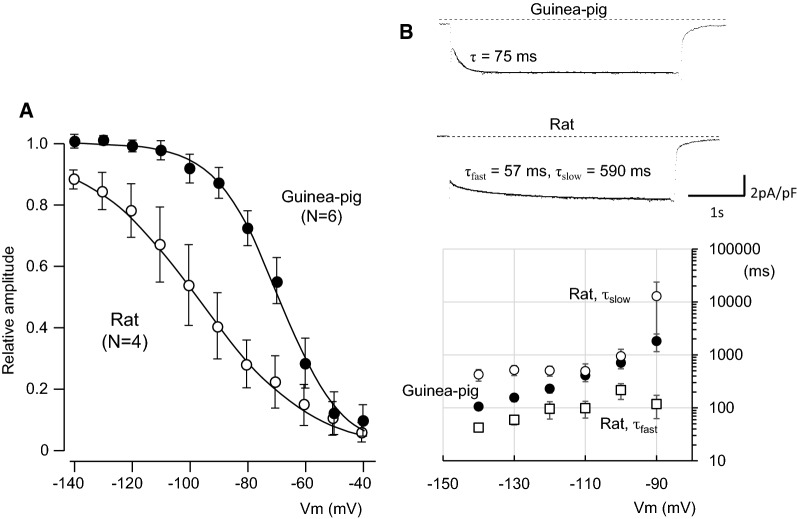
Voltage-dependent kinetics of the I_h_. **A** The steady-state activation curves constructed from rat and guinea pig. Continuous lines are the Boltzmann fits used to determine V_1/2_ and slope factors. **B** Time constants obtained by fitting raw data with a double exponential function in rat, and with one exponential function in guinea pig

The current amplitudes at the beginning and near the end of voltage pulses were measured before and after application of 5 mM Cs^+^ and subsequently 1 mM Cd^2+^, and plotted against the membrane potentials. The *I*_h_ was activated at potentials more negative than − 80 mV in rat and guinea pig PV, and was followed by tail currents upon repolarization to − 40 mV (Fig. 4B). No significant time-dependent current was recorded in rabbit PV cells. The steady-state activation was evaluated by measuring the amplitude of the tail current. The relationship between the test potentials and the relative amplitude of the tail current was fitted with the Boltzmann equation, and plotted in Fig. 5A. The V_1/2_ and slope factor were − 97.3 ± 8.8 and − 16.3 ± 1.1 mV, respectively, in rat PV, − 66.0 ± 3.3 and − 14.9 ± 2.8 mV in guinea pig. The time course of activation from − 140 mV to − 90 mV was analyzed by fitting the time-dependent *I*_h_ with a sum of two exponential functions in rat, and a single exponential function was sufficient for the *I*_h_ of guinea pig PV cells. Thus, the voltage-dependent kinetics were different between rat and guinea pig.

### Comparison of membrane currents blocked by Ba^2+^ in rat, guinea pig, and rabbit

**Fig. 6:**
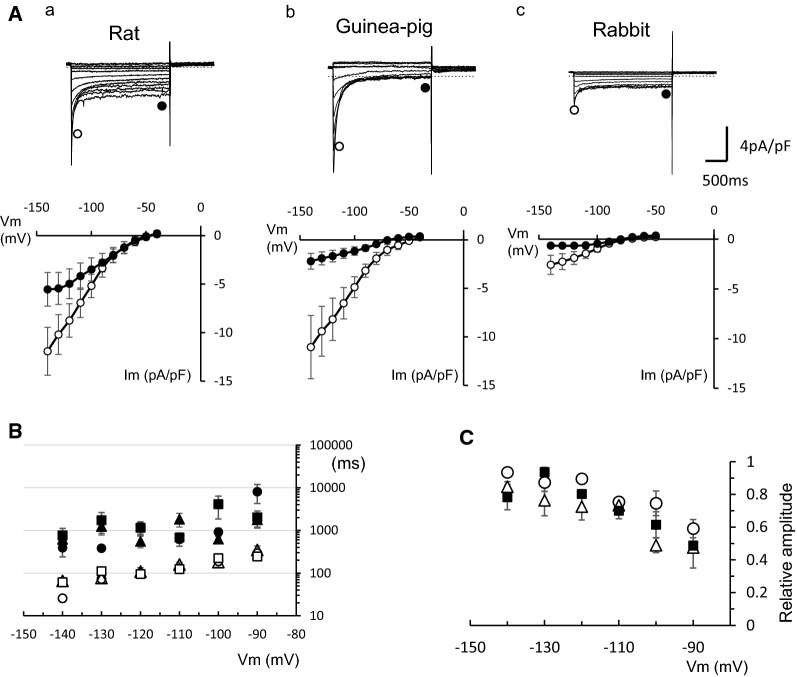
Ba^2+^-sensitive component of the membrane currents in response to 2-s hyperpolarizing voltage pulses from − 40 mV. **A** Representative current traces recorded from PV cells of rat (**a**), guinea pig (**b**), and rabbit (**c**). The currents were obtained by subtracting the currents in the presence of 1 mM Ba^2+^ from those in the absence of Ba^2+^. The *I–V* relationships were obtained for the initial peak and the end of the Ba^2+^-sensitive current. **B** Time constants were obtained by the least squares fit of the Ba^2+^-sensitive current with a sum of two exponential functions. Open and filled symbols indicate fast and slow components, respectively, and circles, squares and triangles indicate rat, rabbit and guinea pig, respectively. **C** Relative amplitude of the fast component. Open circles, open triangles and filled squares indicate rat, guinea pig and rabbit, respectively

We also compared the Ba^2+^-sensitive components among the three species. The Ba^2+^-sensitive components were obtained by subtracting the currents recorded in the presence of 1 mM Ba^2+^ from those in the absence of Ba^2+^. The representative current traces and the current–voltage (*I–V*) relations for the initial peak and near the end of the pulses are shown in Fig. 6A. It was shown that time-dependent decay is marked in rat and guinea pig PV cells, while the decay is only slight in rabbit PV cells. However, the amplitude of steady components seemed larger in rat than in guinea pig. The time course of the current decay was analyzed by the least squares fit with a sum of two exponential functions. The fast and slow components of the time constant and relative weight of fast component are plotted in Fig. 6B, C. The time constants of the current decay were similar among rat, guinea pig, and rabbit.

## Discussion

In the present study, we examined the species difference of the *I*_h_ in PV cardiomyocytes isolated from rat, guinea pig and rabbit. Guinea pig and rat PV cardiomyocytes had a remarkable *I*_h_, and the pharmacological properties and voltage-dependent kinetics were different between the two species. The *I*_h_ of guinea pig was almost completely suppressed by 5 mM Cs^+^, whereas in rats, the *I*_h_ was not suppressed Cs^+^ but by 1 mM Cd^2+^. In the present study, the differences in the *I*_h_ of rat and guinea pig PV cells were distinguished by the different sensitivity to Cs^+^ and Cd^2+^, and ionic selectivity was not examined. It should be noted, however, that in our previous study, the *I*_h_ of rat PV cardiomyocytes was investigated thoroughly in terms of voltage-dependent kinetics, Cl^−^ selectivity, and sensitivity to pH and osmolarity [11]. Furthermore, the *I*_f_ is well known as a Cs^+^-sensitive cation current and its ion selectivity has been extensively examined in sinoatrial node cells [15]. We thus conclude that the *I*_h_ is chiefly due to *I*_f_ in guinea pig, and *I*_Cl,h_ is the major component of the *I*_h_ in rat PV cardiomyocytes. In rabbit PV cardiomyocytes, we failed to detect a slowly activating inward current in response to hyperpolarization. A negligibly small amplitude of the *I*_h_ in rabbit PV cardiomyocytes is not surprising. Although a previous study suggested the existence of *I*_f_ based on sensitivity to Cs^+^, the current amplitude was very small (< 0.6 pA/pF at − 120 mV) [5]. Furthermore, the immunohistochemical study demonstrated that HCN4, a principal isoform underlying sinoatrial *I*_f_, was not expressed in rabbit PV [19]. Thus, the present findings indicate that the ionic nature of the *I*_h_ and its current density are different among rat, guinea pig, and rabbit.

Both the *I*_Cl,h_ of rat and *I*_f_ of guinea pig are characterized as slowly activating inward currents in response to hyperpolarizing pulses, but the voltage-dependent kinetics seem different between the two current systems, as indicated by the V_1/2_ value (− 97.3 mV for the *I*_Cl,h_ of rat and − 66.0 mV for the *I*_f_ of guinea pig). The *I*_f_ activation range comprises the range of diastolic (pacemaker) potentials, and determines the slope of diastolic depolarization in sinoatrial node cells [15]. The V_1/2_ value has been reported to range between − 60 and − 110 mV depending on the experimental condition [15, 20, 21]. Another feature of the *I*_f_ channel is its direct activation by cAMP, which shifts the activation curve toward positive potentials. As for PV cardiomyocytes, Li et al. [17] reported that V_1/2_ of *I*_f_ was approximately − 105 mV in the canine PV myocardium, and shifted to − 87 mV when rapid atrial pacing (at a rate of 800 beats/min) was applied for 10 weeks. It was further shifted to − 69 mV in response to β-adrenoceptor activation. However, the V_1/2_ value of *I*_Cl,h_ has been reported to depend on the intracellular concentration of Cl^−^ ([Cl^−^]_i_). Okamoto et al. [11] reported that the V_1/2_ was − 107.6 mV with 40 mM [Cl^−^]_i_, and − 121.1 mV with 150 mM [Cl^−^]_i_. All these findings together with the present result indicate that the activation range of *I*_f_ is more positive than that of *I*_Cl,h_.

Regarding the molecular nature of *I*_Cl,h_ in rat PV cardiomyocytes, ClC-2 exhibits electrophysiological properties similar to those of *I*_Cl,h_; i.e., a hyperpolarization-activated and slowly activated inward current [22, 23]. ClC-2 belongs to the ClC family, sharing homologous sequence identity [23]. In fact, the electrophysiological properties of the ClC-2 current were remarkably similar to *I*_Cl,h_ [22, 23]. However, Okamoto et al. pointed out several differences between the ClC-2 and *I*_Cl,h_ of rat PV cardiomyocytes, such as the effects of intracellular Cl^−^ and extracellular pH on the steady-state activation, and the responses to changes in extracellular osmolality [11]. Further studies are necessary to clarify these points.

Among the cardiomyocytes isolated from the PV of the three animal species studied, the proportion of spontaneously active cells was highest in rabbit preparations, and rat PV cardiomyocytes did not show spontaneous pacemaker activity in the present study. These species difference might be, in part, explained by the whole-cell IV relationships (Fig. 2), where the amplitude of the inward Ca^2+^ current was smallest in rat, while the membrane potential showing 0 current level was most depolarized in rabbit preparations (approximately  − 40 mV, Fig. 2B). When the Ba^2+^-sensitive current was compared among the three species, the amplitude of the current was lowest in rabbit preparations. The Ba^2+^-sensitive current includes not only *I*_K1_, but also the acetylcholine-activated K^+^ current and other K^+^ currents. However, the major component seemed to be *I*_K1_ because the *I*_K1_ current shows time-dependent decay at strong negative potentials [24, 25], as shown in Fig. 6A, probably because it is blocked by extracellular Na^+^ and possibly by intrinsic mechanisms [24, 25]. Thus, the present findings are in good agreement with a view that the reduced density of *I*_K1_ plays a permissive role in intracellular Ca^2+^-dependent automaticity. Intracellular Ca^2+^ has been reported to be a key factor for the automaticity of the PV cardiomyocytes in various animal species [4, 8, 26–28]. In case of the rat, we have reported that the spontaneous electrical activity was induced by noradrenaline, which activates both α1- and β1-adrenergic receptors to cause Ca^2+^ overload in the sarcoplasmic reticulum, and that it was suppressed by inhibitors of phospholipase C and the inositol 1,4,5-triphosphate receptor [10]. An inhibitor of the Na^+^/Ca^2+^ exchanger, SEA0400, also had inhibitory effects, indicating that the inward current generated by this exchanger contributes to the electrical activity of the PV myocardium. Experimental findings suggesting the importance of intracellular Ca^2+^ dynamics in the spontaneous activity of PV cardiomyocytes have also been reported for guinea pig [29, 30] and rabbit preparations [14, 28]. Under a reduced *I*_K1_ density, even a slight increase in inward current is likely to trigger depolarization of the resting potential in PV cardiomyocytes.

In addition to the low density of *I*_K1_, it has been reported that the densities of the *I*_f_ and the T-type Ca^2+^ current were larger in pacemaking cells than in nonpacemaking cells [5, 31]. Ivabradine, a selective *I*_f_ inhibitor, suppressed the spontaneous activity of rabbit PV cardiomyocytes [32], although ivabradine suppressed not only *I*_f_ but also Ca^2+^ transient. Okamoto et al. showed that Cl^−^ channel blockers attenuated the noradrenaline-induced automaticity in rat PV cardiomyocytes [11]. These findings indicate that the inward current systems, which are activated at the pacemaker range, are more or less able to contribute to spontaneous depolarization under the reduced *I*_K1_ density of PV cardiomyocytes. In the present study *I*_to_ was recorded in 86% of PV cardiomyocytes isolated from rabbit, and the action potential of rabbit PV cardiomyocytes, which had no I_to_, showed that less negative resting membrane and triggered activity was induced by train stimulation at a pacing cycle length of 2 s. This finding is in good agreement with a previous study that showed the amplitude of *I*_to_ varied from cell to cell and was smaller in pacemaking cells than in nonpacemaking cells of rabbit preparations [5].

### Limitations of the present study

PV cardiomyocytes are heterogeneously distributed from the proximity of the LA to the periphery of the PV. They differ in morphology and current densities of individual ionic current systems. In the present study, we only screened several tens of cardiomyocytes in individual animal species; therefore, the number of preparations might not be sufficient to conclude the existence of *I*_Cl,h_ and *I*_f_ in PV cardiomyocytes. In addition, the isolation procedure was slightly different between rabbit and the other two species. We used elastase in addition to collagenase for isolating rabbit PV cardiomyocytes. This is because collagenase alone was not effective to digest the tissue and obtain isolated cardiomyocytes. The possibility that the enzyme treatment might have affected the detection of the *I*_h_ cannot be completely excluded. Future studies that use immunohistochemistry should examine the distribution of *I*_Cl,h_ and *I*_f_ in the PV myocardium.

## Conclusions

In conclusion, the present study showed that the *I*_h_ is chiefly due to *I*_f_ in guinea pig, and *I*_Cl,h_ is the major component of the *I*_h_ in rat PV cardiomyocytes. In rabbit PV cardiomyocytes, the density of the *I*_h_ is negligibly small when compared with rat and guinea pig. Thus, the ionic nature of the *I*_h_ and its current density are clearly different among experimental animal species. These results contribute to our understanding of the cellular mechanism underlying the arrhythmogenicity of PV, and it would be interesting to know the characteristic of the *I*_h_ in human PV cardiomyocytes. Such information would also be useful for drug discoveries that target the pharmacological treatment of atrial fibrillation.

## Data Availability

Not applicable.

## Abbreviations

*I*_h_: Hyperpolarization-activated current
*I*_f_: Hyperpolarization-activated cation current
*I*_Cl,h_: Hyperpolarization-activated Cl^−^ current
I_K1_: Inwardly rectifying K^+^ current

## References

[1] Haïssaguerre M, Jaïs P, Shah DC, Takahashi A, Hocini M, Quiniou G, Garrigue S, Le Mouroux A, Le Métayer P, Clémenty J (1998). Spontaneous initiation of atrial fibrillation by ectopic beats originating in the pulmonary veins. N Engl J Med.

[2] Ye W, Wang J, Song Y, Yu D, Sun C, Liu C, Chen F, Zhang Y, Wang F, Harvey RP, Schrader L, Martin JF, Chen Y (2015). A common Shox2-Nkx2-5 antagonistic mechanism primes the pacemaker cell fate in the pulmonary vein myocardium and sinoatrial node. Development.

[3] Ye W, Song Y, Huang Z, Zhang Y, Chen Y (2015). Genetic regulation of sinoatrial node development and pacemaker program in the venous pole. J Cardiovasc Dev Dis.

[4] Chen YJ, Chen SA (2006). Electrophysiology of pulmonary veins. J Cardiovasc Electrophysiol.

[5] Chen YC, Pan NH, Cheng CC, Higa S, Chen YJ, Chen SA (2009). Heterogeneous expression of potassium currents and pacemaker currents potentially regulates arrhythmogenesis of pulmonary vein cardiomyocytes. J Cardiovasc Electrophysiol.

[6] Takahara A, Sugimoto T, Kitamura T, Takeda K, Tsuneoka Y, Namekata I, Tanaka H (2011). Electrophysiological and pharmacological characteristics of triggered activity elicited in guinea-pig pulmonary vein myocardium. J Pharmacol Sci.

[7] Cheung DW (1981). Electrical activity of the pulmonary vein and its interaction with the right atrium in the guinea-pig. J Physiol.

[8] Hirose M, Laurita KR (2007). Calcium-mediated triggered activity is an underlying cellular mechanism of ectopy originating from the pulmonary vein in dogs. Am J Physiol Circ Physiol.

[9] Maupoil V, Bronquard C, Freslon JL, Cosnay P, Findlay I (2007). Ectopic activity in the rat pulmonary vein can arise from simultaneous activation of alpha1- and beta1-adrenoceptors. Br J Pharmacol.

[10] Okamoto Y, Takano M, Ohba T, Ono K (2012). Arrhythmogenic coupling between the Na^+^-Ca^2+^ exchanger and inositol 1,4,5-triphosphate receptor in rat pulmonary vein cardiomyocytes. J Mol Cell Cardiol.

[11] Okamoto Y, Kawamura K, Nakamura Y, Ono K (2014). Pathological impact of hyperpolarization-activated chloride current peculiar to rat pulmonary vein cardiomyocytes. J Mol Cell Cardiol.

[12] Hamaguchi S, Hikita K, Tanaka Y, Tsuneoka Y, Namekata I (2016). Enhancement of automaticity by mechanical stretch of the isolated guinea pig pulmonary vein myocardium. Biol Pharm Bull.

[13] Chen YJ, Chen SA, Chen YC, Yeh HI, Chan P, Chang MS, Lin CI (2001). Effects of rapid atrial pacing on the arrhythmogenic activity of single cardiomyocytes from pulmonary veins: implication in initiation of atrial fibrillation. Circulation.

[14] Honjo H, Boyett MR, Niwa R, Inada S, Yamamoto M, Mitsui K, Horiuchi T, Shibata N, Kamiya K, Kodama I (2003). Pacing-induced spontaneous activity in myocardial sleeves of pulmonary veins after treatment with ryanodine. Circulation.

[15] Difrancesco D (2010). The role of the funny current in pacemaker activity. Circ Res.

[16] Ehrlich JR, Cha TJ, Zhang L, Chartier D, Melnyk P, Hohnloser SH, Nattel S (2003). Cellular electrophysiology of canine pulmonary vein cardiomyocytes: action potential and ionic current properties. J Physiol.

[17] Li JY, Wang HJ, Xu B, Wang XP, Fu YC, Chen MY, Zhang DX, Liu Y, Xue Q, Li Y (2012). Hyperpolarization activated cation current (I_f_) in cardiac myocytes from pulmonary vein sleeves in the canine with atrial fibrillation. J Geriatr Cardiol.

[18] Ehrlich JR, Cha TJ, Zhang L, Chartier D, Villeneuve L, Hébert TE, Nattel S (2004). Characterization of a hyperpolarization-activated time-dependent potassium current in canine cardiomyocytes from pulmonary vein myocardial sleeves and left atrium. J Physiol.

[19] Yamamoto M, Dobrzynski H, Tellez J, Niwa R, Billeter R, Honjo H, Kodama I, Boyett MR (2006). Extended atrial conduction system characterised by the expression of the HCN4 channel and connexin45. Cardiovasc Res.

[20] Maruoka F, Nakashima Y, Takano M, Ono K, Noma A (1994). Cation-dependent gating of the hyperpolarization-activated cation current in the rabbit sino-atrial node cells. J Physiol.

[21] Shibata S, Ono K, Iijima T (1999). Inhibition by genistein of the hyperpolarization-activated cation current in porcine sino-atrial node cells. Br J Pharmacol.

[22] Duan D, Ye L, Britton F, Horowitz B, Hume JR (2000). A novel anionic Inward rectifier in native cardiac myocytes. Circ Res.

[23] Duan D (2009). Phenomics of cardiac chloride channels: the systematic study of chloride channel function in the heart. J Physiol.

[24] Sakmann B, Trube G (1984). Voltage-dependent inactivation of inward-rectifying single-channel currents in the guinea-pig heart cell membrane. J Physiol.

[25] Shieh RC (2000). Mechanisms for the time-dependent decay of inward currents through cloned Kir2.1 channels expressed in Xenopus oocytes. J Physiol.

[26] Henry AD, MacQuaide N, Burton FL, Rankin AC, Rowan EG, Drummond RM (2018). Spontaneous Ca^2+^ transients in rat pulmonary vein cardiomyocytes are increased in frequency and become more synchronous following electrical stimulation. Cell Calcium.

[27] Pasqualin C, Yu A, Malécot CO, Gannier F, Cognard C, Godin-Ribuot D, Morand J, Bredeloux P, Maupoil V (2018). Structural heterogeneity of the rat pulmonary vein myocardium: Consequences on intracellular calcium dynamics and arrhythmogenic potential. Sci Rep..

[28] Chang SH, Chen YC, Chiang SJ, Higa S, Cheng CC, Chen YJ, Chen YJ, Chen SA (2008). Increased Ca^2+^ sparks and sarcoplasmic reticulum Ca^2+^ stores potentially determine the spontaneous activity of pulmonary vein cardiomyocytes. Life Sci.

[29] Namekata I, Tsuneoka Y, Takahara A, Shimada H, Sugimoto T, Takeda K, Nagaharu M, Shigenobu K, Kawanishi T, Tanaka H (2009). Involvement of the Na^+^/Ca^2+^ exchanger in the automaticity of guinea-pig pulmonary vein myocardium as revealed by SEA0400. J Pharmacol Sci.

[30] Tanaka Y, Obata K, Ohmori T, Ishiwata K, Abe M, Hamaguchi S, Namekata I, Tanaka H (2019). Angiotensin II induces automatic activity of the isolated guinea pig pulmonary vein myocardium through activation of the IP_3_ receptor and the Na^+^-Ca^2+^ exchanger. Int J Mol Sci.

[31] Chen YC, Chen SA, Chen YJ, Tai CT, Chan P, Lin CI (2004). T-type calcium current in electrical activity of cardiomyocytes isolated from rabbit pulmonary vein. J Cardiovasc Electrophysiol.

[32] Suenari K, Cheng CC, Chen YC, Lin YK, Nakano Y, Kihara Y, Chen SA, Chen YJ (2012). Effects of ivabradine on the pulmonary vein electrical activity and modulation of pacemaker currents and calcium homeostasis. J Cardiovasc Electrophysiol.

